# Morse Taper Connection Implants Placed in Grafted Sinuses in 65 Patients: A Retrospective Clinical Study with 10 Years of Follow-Up

**DOI:** 10.1155/2017/4573037

**Published:** 2017-08-07

**Authors:** Francesco Mangano, Renata Bakaj, Irene Frezzato, Alberto Frezzato, Sergio Montini, Carlo Mangano

**Affiliations:** ^1^Department of Medicine and Surgery, University of Insubria, 21100 Varese, Italy; ^2^Private Practice, 45100 Rovigo, Italy; ^3^Private Practice, 22019 Tremezzo, Italy; ^4^Department of Dental Science, Vita Salute S. Raffaele University, 20132 Milan, Italy

## Abstract

**Purpose:**

To investigate the 10-year survival and complication rates of Morse taper connection implants (MTCIs) placed in grafted sinuses.

**Methods:**

This study reports on patients treated with maxillary sinus augmentation (with the lateral window technique (LWT) or the transalveolar osteotomy technique (TOT)) and installed with MTCIs supporting fixed restorations (single crowns (SCs) and fixed partial dentures (FPDs)), in two dental clinics. The outcomes of the study were the 10-year implant survival and complication rates.

**Results:**

Sixty-five patients (30 males and 35 females) with a mean age of 62.7 (±10.2) years were installed with 142 MTCIs: 79 fixtures were inserted with the LWT and 63 were placed with the TOT. After ten years, five implants failed, for an overall survival rate of 96.5%. Three implants failed in the LWT group, for a survival rate of 96.3%; two implants failed in the TOT group, for a survival rate of 96.9%. The 10-year incidence of biologic complications was 11.9%. Prosthetic complications were all technical in nature and amounted to 7.6%.

**Conclusions:**

MTCIs seem to represent a successful procedure for the prosthetic restoration of the grafted posterior maxilla, in the long term. This study was registered in the ISRCTN registry with number ISRCTN30772506.

## 1. Introduction

In the posterior maxilla, sinus pneumatisation with ageing [1] and postextraction alveolar crest resorption [2] can severely affect the amount of bone volume, jeopardizing a successful osseointegration, unless a reconstructive osseous surgery is performed to sustain a functional and aesthetic implant-supported restoration [3].

Currently, bone volume increase in the posterior maxilla is mainly obtained by maxillary sinus floor augmentation [4–6]. This surgical procedure was found to be reliable and it can be performed according to two major techniques: the lateral window approach [7], which is still the most common method, and the transalveolar osteotomy technique [8, 9].

Many variables should be taken into consideration by the clinician before choosing the surgical technique, such as the residual bone quantity [9], the type of grafting material [10–12], the use of barrier membranes [13], the implant insertion timing in relation to grafting (one- or two-stage approach) [14, 15], and the type of implants to be placed. The one-stage approach consists of simultaneous implant placement into the augmented sinus graft [14], while the two-stage method involves implant insertion secondary to reconsolidation of the bone graft [15].

Morse taper connection implants (MTCIs) represent a valid treatment option for restoring partially and completely edentulous patients, as demonstrated by several long-term follow-up studies [16–19].

In MTCIs, the implant-abutment connection relies on the “cold welding” achieved through frictional resistance between the surfaces of the abutment and the implant [18, 20]. If the taper angle is less than 2°, the connection is called “self-locking” [17, 20].

Although several studies have confirmed that the use of MTCIs yields excellent survival and success rates [16–19, 21–23], there are currently no clinical studies on the long-term outcomes of MTCIs placed in the grafted sinuses.

In light of the above, the purpose of this retrospective clinical study was to investigate the 10-year survival and complication rates of MTCIs placed in grafted sinuses via the lateral window technique or the transalveolar osteotomy technique.

## 2. Materials and Methods

### 2.1. Patient Population

We conducted a retrospective clinical study on patients that have been treated with maxillary sinus augmentation (with the lateral window or the transalveolar osteotomy technique) and with fixed prosthetic restorations (SCs and FPDs) supported by MTCIs, in the period from January 2003 to August 2006, in two private dental clinics (located in Gravedona, Como, Italy, and in Padua, Italy, resp.).

Patients selected for the present study were identified through the records of two dental clinics; these records included all information about each enrolled patient (patient-related information: systemic health, age at surgery, gender, smoking habit, and oral hygiene) and each implant-supported restoration placed (implant-related information: position, premolar or molar; length and diameter; restoration-related information: type of prosthesis, SC or FPD; date of deliveries). The customized records included all information about any implant failure and/or biological/prosthetic complication that occurred during the 10-year follow-up.

Patients were excluded from the present retrospective study in case of (1) systemic diseases or ongoing treatments/conditions that may contraindicate intervention (uncontrolled diabetes, immunocompromised states, chemo/radiotherapy of the head/neck region, treatment with amino-bisphosphonates, psychiatric disorders, and abuse of drugs/alcohol); (2) oral diseases (nontreated periodontal disease and active/chronic/persistent sinus infections); (3) nonacceptance or inability to attend the 10-year follow-up clinical/radiographic examination for different reasons (death, hospitalization, and transferring to another country or city).

All of the enrolled patients were requested to return to the dental clinic and to attend a 10-year control follow-up clinical/radiographic examination. Patients who did not accept to attend the 10-year follow-up control, as well as patients who could not attend it, were excluded from the present study. All included patients read and signed a written consent form for inclusion in this retrospective study. Approval of the Ethics Committee at University of Insubria was obtained for this study; the Helsinki Declaration of 1975, as revised in 2008, was followed. In addition, the study was registered in the publicly available ISRCTN clinical studies registry, a trial registry recognized by the WHO, with number ISRCTN30772506.

### 2.2. Implant Design and Surface Characterization

**Figure 1:**
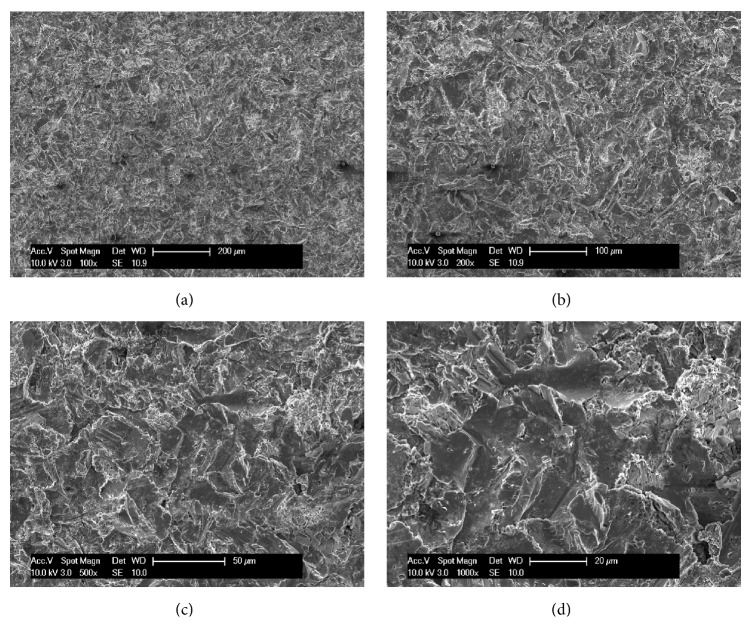
The sandblasted-acid-etched surface of the implants used in this study, at different magnification: (a) ×100; (b) ×200; (c) ×500; (d) ×1000. Implant surface was treated with a sandblasting process producing an average roughness *R*_*a*_ of 2.5 *μ*m: fixtures were blasted with alumina particles. Sandblasting was followed by a decontamination treatment series, including a passivation process with nitric acid.

**Figure 2:**
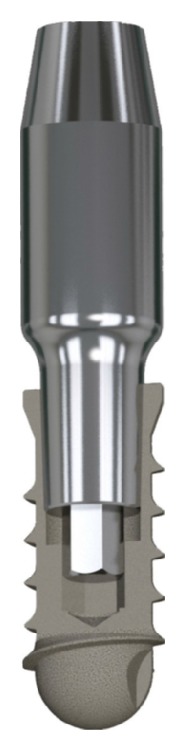
The implants used in this study featured a cone Morse taper interference-fit (TIF) locking-taper, with a taper angle of 1.5°, combined with an internal hexagon.

The implants used were screw-shaped and made of grade-5 titanium alloy (Leone Implants®, Florence, Italy). Their surfaces were blasted with 350 *μ*m Al_3_O_2_ particles and acid-etched with HNO_3_, producing a *R*_*a*_ value (the peak-valley distance of surface irregularities) of 2.5 *μ*m [24] (Figure 1). The implant-abutment connection is based on a Morse taper with an angle of 1.5° combined with an internal hexagon [16–19, 21] (Figure 2).

### 2.3. Preoperative Work-Up

Each patient underwent a primary investigation within a complete medical examination of the hard and soft oral tissues and panoramic radiographs. Where needed, computed tomography (CT) scans were requested, in selected patients. CT datasets were acquired and then converted into DICOM format. DICOM files were used to obtain a three-dimensional reconstruction of the jaws in implant navigation software, which showed the anatomic tissues including residual bone volume, thickness/density of the cortical and cancellous bone, ridge angulations, and also possible sinus pathology. Each implant site was carefully assessed. An accurate evaluation of the edentulous ridges using casts and diagnostic wax-up were included in the preoperative workups.

### 2.4. Surgery

Patients were instructed to rinse with 0.2% chlorhexidine mouthwash (Chlorhexidine®; OralB, Boston, MA) for 1 minute twice daily, two days before surgery, and also for 1 minute prior to the surgery. All patients received prophylactic antibiotic therapy of 2 g of amoxicillin + clavulanic acid 1 hour before the surgery. After surgery, they continued taking antibiotics twice daily for 6 days. All patients were treated under local anaesthesia using 4% articaine with adrenaline 1 : 100000.

When the lateral window technique (LWT) was used, the surgeon proceeded as follows. In order to expose the maxillary sinus lateral side, a horizontal crestal incision and two vertical incisions were performed in the buccal mucosa, in order to raise a mucoperiosteal flap. Using piezosurgery equipment under continuous saline irrigation, it was possible to outline a bone window approximately 1.5 × 1.5 cm in size. The sinus mucosa was separated from the bony surface of the sinus floor with an elevator and the bony window fragment removed. Great effort was made to prevent disruption of the Schneiderian membrane; when this occurred, a collagen barrier was used to contain the graft. After the elevation of the Schneiderian membrane was completed, the gap created between the maxillary alveolar process and the new sinus floor was filled with coral-derived porous hydroxyapatite (Biocoral®, Biocoral Inc., Saint Gonnery, France) blocks. These blocks were shaped and modelled by the surgeon, who also used porous hydroxyapatite granules to completely fill in the spaces between the porous material blocks and residual bone crest. The granules were interspersed with tetracycline powder to obtain a local antibiotic effect and moistened with physiological saline solution so that this mixture could be easily moulded to fit the gaps. The sinus window was then sealed with the bony window fragment, covered by a collagen membrane and the mucosa sutured with non-absorbable sutures. When using a two-stage approach, the healing period for grafted sinuses was 6 months before implant placement. Conversely, in the one-stage approach, simultaneous implant insertion was performed. Implant placement was performed as follows. Spiral drills of increasing diameter were used under constant irrigation, to prepare the implant site. All implants were placed at the bone crest level.

When the transalveolar osteotomy technique (TOT) was used, the surgeons proceeded as follows. A horizontal crestal incision with minimal lateral releases was performed to expose all implant sites. A mucoperiosteal flap was elevated. The preparation of the site was performed with a speed reducing gear handpiece under copious saline irrigation. Using the aforementioned drill sequence, the palatal osseous lid was removed and the Schneiderian membrane was meticulously lifted by means of the sequential use of osteotomes and a metal mallet. After the elevation was completed, the sinus cavity was grafted with coral-derived hydroxyapatite granules, mixed with tetracycline powder. The material was packed into the cavity and the implant was placed. The fixture was tightly screwed by means of a hand ratchet until it came into alignment with the crest of alveolar bone. Excessive graft material particles were removed and the flap was repositioned. Primary interrupted tension-free wound closure was accomplished with nonabsorbable sutures. With the transalveolar osteotomy technique, the implants were submerged for a minimum healing period of 3 months before beginning the prosthetic phases.

### 2.5. Healing Period, Second-Stage Surgery, and Prosthetic Restoration

Postoperative pain was controlled in all patients with 100 mg nimesulide intake every 12 hours for 2 days and detailed oral hygiene instructions were given, including mouth rinses with 0.2% chlorhexidine for 7 days. Sutures were removed around 8–10 days after the surgery.

The submerged healing period lasted around 3–9 months (lateral window technique, two-stage approach = 6 + 3 months; lateral window technique, one-stage approach = 3 months; transalveolar osteotomy technique = 3 months). A second surgery was performed to accede to the healed implants and to place the healing abutments. After two weeks, impressions were taken and, one week later, the provisional restorations were provided. The provisional restorations remained in situ for 3 months, before placing definitive restorations. All definitive restorations (SCs and FPDs) were ceramometallic and cemented with a temporary oxide-eugenol cement.

### 2.6. Implant Survival and Complications

Implants were classified as “surviving” when still functioning at the final follow-up.

Conversely, all implants that were lost and/or had to be removed (for implant mobility due to absence and/or loss of osseointegration in absence of infection, for recurrent/persistent peri-implantitis, and for implant body fracture) were considered as “failed.”

In addition, all biologic and prosthetic complications registered during the entire follow-up period were considered. Among the biologic complications, loss of the graft, sinus infection, peri-implant mucositis, and peri-implantitis were considered [25]. Among the prosthetic complications, all mechanical complications (i.e., complications affecting the prefabricated implant components at the implant-abutment interface such as abutment loosening and abutment fracture) and all technical complications (i.e., complications affecting the superstructures made by the dental technician, such as loss of retention, ceramic chipping/fracture, and fracture of the metallic framework of restoration) were considered [26].

All data were carefully analysed in a statistical software package. Means and standard deviations, ranges, and confidence intervals were calculated for the available quantitative variables (patients' age). Absolute and relative frequency distributions were calculated for all the available qualitative variables. The distribution of the patients (by gender, age at surgery, smoking, and oral hygiene habits) and the distribution of the implants (by sinus augmentation technique, position, length and diameter, and type of supported restoration) were investigated, and a Chi-square test (with level of significance set at 0.05) was used to calculate the differences in distribution between the groups. Finally, implant survival and complications were calculated using the implant as a statistical unit.

## 3. Results

### 3.1. Patients Enrolled and Implants Placed

Sixty-five patients were enrolled in this study: 30 males (30/65: 46.2%) and 35 females (35/65: 53.8%) with an average age of 62.7 ± 10.2 years (median 66, range 38–79, 95% CI: 60.3–65.1). Most of the enrolled patients (42/65 patients, 64.6%) were between the ages of 60 and 79at surgery, whereas 21 (21/65, 32.3%) were between the ages 40 and 59 and only two patients (2/65: 3.1%) were between the ages of 20 and 39 years. Fifteen patients (15/65: 23.1%) were smokers. Among all patients, 35 (35/65: 53.8%) had satisfactory oral hygiene with low plaque score levels and 30 patients (30/65: 46.2%) had unsatisfactory oral hygiene levels. The distribution of the patients by gender, age at surgery, smoking habit, and oral hygiene is reported in Table 1.

As twelve patients required bilateral maxillary sinus augmentation, the number of sinus augmentation procedures amounted to 77. Forty-five of these procedures were performed with the lateral window technique and 32 were performed with the transalveolar osteotomy technique.

In total, 142 implants were placed: 79 (79/142: 55.6%) were inserted with the lateral window technique and 63 (63/142: 44.4%) were placed with the transalveolar osteotomy technique. With regard to the distribution of the implants, 55 (55/142: 38.7%) were premolars and 87 (87/142: 61.3%) were molars; the most frequent length was 10 mm (49/142 fixtures, 34.5%), followed by 8 mm (35/142 implants, 24.7%), 12 mm (32/142 implants, 22.5%), and 14 mm (26/142 implants, 18.3%). The most frequently used diameter was 4.1 mm (75/142 fixtures, 52.8%), followed by 4.8 mm (43/142 fixtures, 30.3%) and 3.3 mm (24/142 fixtures: 16.9%). Finally, with regard to the prosthetic restoration, as 44 fixtures were used to support SCs, and 98 fixtures were used to support FPDs, the final prosthetic restorations amounted to 44 SCs and 47 FPDs (43 FPDs were supported by two implants and 4 FPDs were supported by three implants, resp.). The distribution of the fixtures by surgical technique, position, length, diameter, and type of supported restoration is reported in Table 2.

### 3.2. Implant Survival and Complications

**Figure 3:**
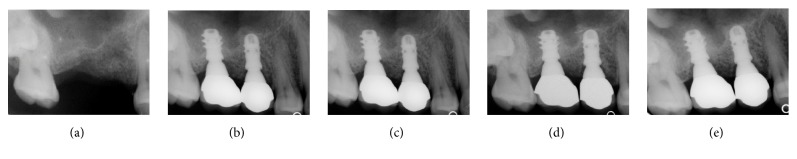
Two implants (#15 and #16) inserted with the transalveolar osteotomy technique: (a) preoperative rx; (b) radiographic control at the delivery of final restorations; (c) radiographic control 1 year after implant placement; (d) radiographic control 5 years after implant placement; (e) radiographic control 10 years after implant placement.

**Figure 4:**
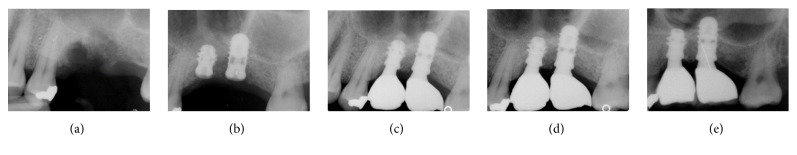
Two implants (#25 and #26) inserted with the transalveolar osteotomy technique: (a) preoperative rx; (b) the implants placed after the sinus elevation with the Summers technique; (c) radiographic control 1 year after implant placement; (d) radiographic control 5 years after implant placement; (e) radiographic control 10 years after implant placement.

**Figure 5:**
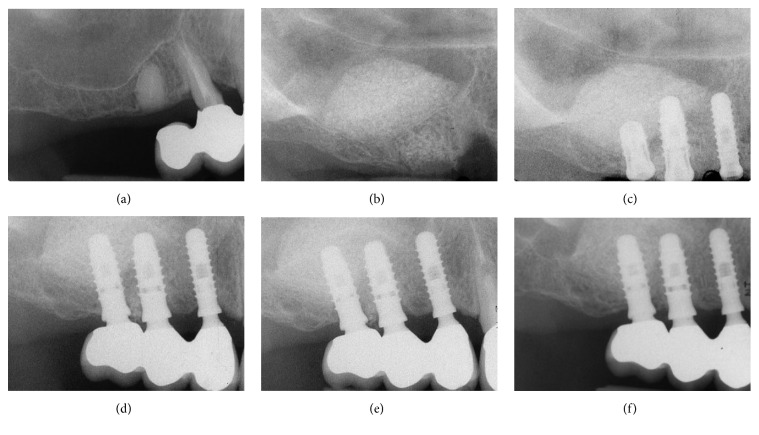
Three implants (#14, #15, and #16) inserted with the lateral window technique. (a) preoperative rx; (b) periapical rx after the sinus augmentation procedure according to Tatum; (c) 6 months later the implants are inserted; (d) radiographic control at the delivery of the final restoration; (e) radiographic control 5 years after implant placement; (f) radiographic control 10 years after implant placement.

At the end of the study, 10 years after implant placement, only five implants failed (5/142), for an overall survival rate of 96.5% (Figures 3–5). Three implants failed in the lateral window group (3/79), for a survival rate of 96.3%. Two implants failed in the transalveolar osteotomy group (2/63), for a survival rate of 96.9%. Three of the failed implants were removed during the second-stage surgery, because they showed clinical mobility due to absence of osseointegration. These failures occurred before the connection of the prosthetic abutment and were therefore defined as “early” failures. Conversely, two implants failed in the same patient due to recurrent peri-implant infection and were removed due to massive bone loss 6 years after placement. All information regarding the failed implants is summarized in Table 3.

With regard to biologic complications, one patient experienced infection and loss of the graft after sinus augmentation with the lateral window technique, probably due to an undetected perforation of the Schneiderian membrane. This sinus was surgically revisited and cleaned. This intervention was followed by a prolonged systemic antibiotic treatment and a healing period of 6 months and subsequent successful augmentation. Conversely, no biologic complications were found for the implants placed according to the transalveolar osteotomy technique.

In addition, nine implants (9/142: 6.3%) suffered from a reversible inflammation of the peri-implant soft tissues (peri-implant mucositis) with exudation and discomfort, but without radiographic evidence of bone loss. Eight implants (8/142: 5.6%) suffered from infection of the hard and soft tissues (peri-implantitis) with associated peri-implant marginal bone loss. Among these implants, however, only two were lost due to untreatable, recurrent peri-implantitis with advanced bone loss; the other five implants were treated with professional oral hygiene and in these cases failure was avoided. Overall, the 10-year incidence of biologic complications affecting implants was 11.9%.

Finally, with regard to prosthetic complications, no mechanical (i.e., at the implant-abutment interface) complications were registered; however, seven restorations (4 SCs and 3 FPDs) experienced ceramic chipping/fractures, which required intervention from the dental technician. The prosthetic complications amounted to 7.6% (7/91 prosthetic restorations).

## 4. Discussion

It has been broadly proven that maxillary sinus augmentation is a highly successful and predictable method of obtaining sufficient bone height for posterior maxillary implant placement [3–6, 10].

In an interesting systematic review, which included studies with at least 3 years of follow-up, 18 articles for the LWT (6,500 implants in 2,149 patients) and 7 for the TOT (1,257 implants in 704 patients) were selected [5]. The overall implant survival was 93.7% and 97.2% for the LWT and the TOT, respectively [5].

These outcomes were confirmed by more recent reviews of the current literature [3, 4]. In fact, Duttenhoefer et al. conducted a meta-analysis to study the influence of various treatment modalities (surgical technique, timing of implant placement, grafting materials, and use of membranes) on the implant survival in the grafted maxillary sinus [3]. This review included 122 publications on 16268 dental implants inserted in grafted sinuses [3]. At the end of this work, no differences were found in the implant survival with respect to each surgical approach, grafting material and implant type. However, the application of membranes showed a positive influence on the long-term implant outcomes, independently of other cofactors [3].

In this retrospective study, we have evaluated the 10-year implant survival and complication rates of MTCIs placed in grafted sinuses using two different surgical techniques (the LWT or the TOT). In accordance with the aforementioned literature, a satisfactorily high implant survival rate was found for both LWT (96.3%) and TOT (96.9%).

Different clinical studies have suggested that autogenous bone is the best reconstructive material, because of its osteogenic, osteoconductive, and osteoinductive properties [27, 28].

However, in recent clinical studies, bone substitutes such as allogeneic [29], xenogenic [11], and synthetic grafts [30, 31] and composite materials [32] have also been successfully employed in maxillary sinus augmentation.

Starch-Jensen et al. found that the 5-year implant survival rate after sinus elevation with autogenous bone graft or bovine bone mineral was 97% and 95%, respectively [4], and the reduction in vertical height of the augmented sinus with the two materials was the same. In this review, similarly high survival rates were found for implants, regardless of the grafting material used [4]. High implant stability, high patient satisfaction, and limited peri-implant marginal bone loss were found [4].

In another review of the literature, Danesh-Sani et al. confirmed that bone substitutes (allografts, xenografts, and synthetic materials) were good alternatives to autogenous bone, avoiding the disadvantages related to autografts (morbidity rate and limited availability) [10].

Here, we used a coral-derived porous hydroxyapatite for maxillary sinus augmentation. In accordance with a previous report [30], the present study has noted excellent results with the use of coralline calcium phosphates for grafting of the maxillary sinus.

It must be pointed out that, recently, the role and the importance of the grafting material has been partially revisited [33]. In a review on clinical studies with a follow-up period of 48 to 60 months, the implant survival rate was 99.6% for surgeries conducted with graft material and 96% for surgeries performed without it [33]. These results suggest that sinus lift can be a safe and predictable treatment procedure with low complication rates, irrespective of the use of biomaterials [33].

Recent studies have reported excellent survival and success rates for sinus grafting and implant placement in both one- and two-stage protocols [14, 15].

A noteworthy systematic review revealed that the placement of implants in combination with sinus elevation is a predictable procedure, showing high implant survival rates with low incidence of complications [6].

Once again, our present study seems to be in accordance with the current literature. In fact, excellent survival rates were found with the LWT, with both staged and simultaneous implant placement.

The choice of simultaneous implant placement and grafting procedure is generally highly influenced by the residual crestal bone height, which must be sufficient to provide adequate primary implant stability [9]. A recent literature review investigated the correlation between the amount of remaining crestal alveolar bone before sinus augmentation and implant survival. The findings indicated that a residual bone height of less than 4 mm may influence the survival/success rates of fixtures placed in combination with sinus elevation using osteotomes [9].

Comparable studies obtained findings that support a positive influence of rough surfaces on osseous integration in the posterior maxilla [34].

In a recent systematic review for implant survival in maxillary sinus augmentation, implants with rough surfaces displayed a higher survival rate (97.6%; 95% CI: 96.7–98.5%) than implants with machined surfaces (89.4%; 95% CI: 83.0–95.8%), within no correlation or influence from the graft type [35].

These results were also confirmed by a previous review of the literature, in which dental implants placed in the posterior augmented maxilla showed an average survival rate of 92.6% [36]. The use of rough-surfaced implants and particulate bone resulted in an increased implant survival rate (94.5%) and the use of a membrane to cover the graft increased the survival rate to 98.6% [36].

In the present study, in accordance with the aforementioned research, the use of sandblasted MTCIs guaranteed excellent implant survival and success rates. Moreover, only a few biologic (11.9%) and prosthetic (7.6%) complications were reported in our present long-term retrospective study.

All implants with screw type connections show a microgap of variable dimensions (40–100 *μ*m) at the interface between the implant and the abutment [37]. Scientific evidence suggests that bacterial leakage and colonization of this microgap may be responsible for inflammatory cell recruitment and activation at the corresponding bone level, causing the development of marginal bone loss [37].

Provided that the absence of the microgaps is associated with reduced inflammation and bone loss, an efficient seal against microbial penetration may be provided by MTCIs [20, 38]. Indeed, this screwless connection reduces the microgap (1–3 *μ*m) dimensions at the implant-abutment interface with a tight closure against the fixture; thus it contributes to a minimal level of peri-implant inflammation [20, 38].

In addition, no prosthetic complications were reported at the implant-abutment interface in our present study. This is similar to results from previous studies on MTCIs [16–20].

The stability of the implant-abutment connection is key for the long-term success of an implant-supported prosthetic restoration [16–19]. In addition, it may contribute to a more favourable load distribution into the bone [20, 39] and therefore to a reduction of the marginal bone loss around implants in the long term. This hypothesis needs further investigation, but, if correct, MTCIs may reduce micromovements at the implant-abutment interface, preventing crestal bone loss [39].

Moreover, MTCIs inherently have “platform switching” [40]. With platform switching, any potential microgap between the implant and the abutment (which harbours bacteria, responsible for toxin production) is displaced horizontally and away from the bone, with the possibility of reducing inflammation and of minimizing bone loss [40]. This aspect may further improve the long-term outcomes of Morse taper connection implants, reducing the incidence of biologic complications. In addition, a larger space exists for the organization of thick soft tissues, that can further protect the bone from resorption [40].

Our present study has limits. First, it is a retrospective clinical study, and the retrospective design is not the best way to investigate the long-term outcomes of dental implants (in fact, a prospective study design would be preferable, but the best solution to draw more specific conclusions about a treatment procedure would certainly be a randomized clinical trial). Second, our conclusions are based on a limited number of patients. Further, long-term prospective clinical studies (or even better, randomized clinical trials) on a larger sample of patients are therefore needed, to confirm the positive outcomes emerging from our present clinical study.

## 5. Conclusions

Within the limits of the present clinical study (retrospective design and limited number of patients enrolled), it can be stated that MTCIs represent a successful procedure for the prosthetic restoration of the grafted posterior maxilla, with both LWT and TOT, in the long term. In fact, a 10-year overall implant survival rate of 96.5% was found. Three implants failed in the lateral window group (3/79), for a survival rate of 96.3%, and two implants failed in the transalveolar osteotomy group (2/63), for a survival rate of 96.9%. A low incidence of biologic complications was reported in this study, in the long term (11.9%). In addition, the high mechanical stability of MTCIs likely contributed to the limited amount of prosthetic complications observed in this study (7.6%).

## Figures and Tables

**Table 1:** Patient distribution.

	Number of patients (%)	^*∗*^ *p*
Gender		
*Males*	30 (46.2%)	0.535
*Females*	35 (53.8%)	
Age at surgery		
*20–39 years*	2 (3.1%)	<0.0001
*40–59 years*	21 (32.3%)	
*60–79 years*	42 (64.6%)	
Smoking habit		
*Yes*	15 (23.1%)	<0.0001
*No*	50 (76.9%)	
Oral hygiene		
*Satisfactory*	35 (53.8%)	0.535
*Not satisfactory*	30 (46.2%)	
Total	65 (100%)	—

^*∗*^ *p* = Chi-square test.

**Table 2:** Implant distribution.

	Number of implants (%)	^*∗*^ *p*
Sinus augmentation technique		
*Lateral window technique*	79 (55.6%)	0.179
*Transalveolar osteotomy technique*	63 (44.4%)	
Position		
*Premolars*	55 (38.7%)	0.007
*Molars*	87 (61.3%)	
Length		
*8 mm*	35 (24.7%)	0.045
*10 mm*	49 (34.5%)	
*12 mm*	32 (22.5%)	
*14 mm*	26 (18.3%)	
Diameter		
*3.3 mm*	24 (16.9%)	<0.0001
*4.1 mm*	75 (52.8%)	
*4.8 mm*	43 (30.3%)	
Restoration		
*SC*	44 (31.0%)	<0.0001
*FPD*	98 (69.0%)	
Total	142 (100%)	—

^*∗*^ *p* = Chi-square test.

**Table 3:** Failed implants.

Gender	Age	Smoke	Hygiene	Procedure	Position	Type	Reason/timing
Male	46	No	Poor	LWT	Premolar	4.1 × 10	Failure to osseointegrate after 3 months
Male	66	Yes	Good	LWT	Premolar	4.1 × 10	Failure to osseointegrate after 3 months
Female	59	No	Good	LWT	Molar	4.8 × 8	Failure to osseointegrate after 3 months
Female	66	Yes	Poor	TOT	Premolar	4.1 × 12	Peri-implantitis after 6 years
Female	66	Yes	Poor	TOT	Molar	4.8 × 10	Peri-implantitis after 6 years
